# Supplementary material for: The Role of Cognitive Reserve in Post-Stroke Rehabilitation Outcomes: A Systematic Review
Source: Brain Sci. 2024 Nov 15;14(11):1144. doi: 10.3390/brainsci14111144 (PMC11591582; doi:10.3390/brainsci14111144)
Supplement: Supplementary file 1 [file brainsci-14-01144-s001.zip › brainsci-3276919-supplementary.pdf]

Supplementary Materials

Table S1. Characteristics of the included studies.

Author	Year	State	Sample	Inclusion Criteria	CR Proxies	Outcome Measure	Results	Conclusion
Abdullah	2021	Asia	N=80<br><br>Ischemic stroke	- diagnoses of stroke;<br>- age between 17 and 70;	CRIq<br>Education	- CRIq-M;<br>- (Malaysian DASS-21);<br>- WHOQOL BREF-2;<br>- MoCA<br>- -mRS.	CR is positively correlated with cognitive function. Education was a significant predictor of cognitive impairment whereas cognitive impairment, as assessed by the MoCA, is a significant predictor for cognitive reserve in people with stroke.	.
Alladi	2015	Asia	N= 608<br><br>Ischemic stroke	- ischemic stroke<br>- >18 years<br>- evaluated 3 to 24 months after stroke<br>- no disabling stroke (mRS score<4)<br>- no severe comorbidities<br>- adequate data<br>- no preexisting dementia	- Bilingualism	- ACE-R<br>-Clinical Dementia Rating scale	Bilingualism is an independent predictor of poststroke cognitive impairment.	
Bartfai	2022	EU	N=59<br><br>Stroke or TBI	In- or outpatients (n = 59) with mild to moderate stroke or traumatic brain injury.	- Premorbid occupation<br>- Education level	-<br>BI<br>- Matrices (WAIS-III)<br>- Albert's Test<br>- RBMT ,<br>-HADS<br>-APT test	Stroke patients benefited from APT training. Cognitive reserve was associated	

							with change and APT as the sole pre-injury factor.
Basagni	2023	EU	N=27<br><br>TBI, ischemic or hemorrhagic stroke, anoxic damage, and other pathologies	- Being part of the sub-group of patients of Study 1<br>-LCF 6<br>- first admission to a rehabilitation center after an acute neurological event With GCS score <9 for more than 24h	- CRIq<br>- DRS<br>- LCF	- Tele-GEMS<br>- GOS-E	Higher levels of CR and LCF significantly predicted cognitive performance 4 years later. However, CR did not predict functional outcome, which was only predicted by lower disability scores at discharge.
Bertoni	2020	EU	N=94<br><br>TBI, ischemic or hemorrhagic stroke, and anoxic damage	- First admission after a neurological event causing altered consciousness, with aGCS score <9 for over 24 hours<br>- No prior neurological or psychiatric history<br>- Italian native speaker, aged 18-70 on admission<br>- Caregiver presence<br>- Stay uninterrupted for more than 20 days	-CRIq (administered to the caregiver)	-Group 1 with LCF > 5: neuropsychological (NPS) assessment on admission (T1). Before discharge (T2), a follow-up NPS test was performed, administering the LCF, DRS and mBI scales.<br><br>-Group 2 with LCF<6: LCF, DRS and mBI tests on admission and at discharge (T1 and T2).	Patient with high cognitive reserve demonstrated significant improvement in DRS score at discharge. Earlier rehabilitation led to greater improvement in part B of the TMT.
Dekhtyar	2020	EU	N=3109<br><br>Stroke, TBI, brain tumor	- 36 with Neurological events (aphasia BAA and MAA and BHA and MHA were matched	Bilingualism	Cognitive assessment at baseline:<br>- (LUQ, only Bilingual	No bilingual cognitive control advantage on reaction times

				on age and education) at least 6-months post injury.<br>- no pre-morbid psychiatric or neurologic illness.<br>-		participants);<br>- (BNT e BAT) Part B.<br>- NLTT =<br>- All participants completed a non-linguistic cognitive control task that included congruent and incongruent conditions	in healthy adult groups. BAA were faster than MAA, suggesting that bilingualism may contribute to cognitive reserve in adults with aphasia.
Durrani	2021	Canada	N=10916<br><br>Vascular brain injury (nonlacunar infarcts or high WMH).	No prior history of stroke, dementia, or other CNS diseases	- Education<br>- involvement in social activities<br>- marital status<br>- height<br>- leisure physical activity	-Medical history, demographics, lifestyle, anthropo-metric measures, cognition, and MRI were recorded.<br>-MoCA<br>-WAIS -IVDSST).	~~Factors like~~ Education, physical activity, marital partnership, height, and social participation are linked to higher cognition. Vascular brain injury is associated with lower cognition, and this association is not influenced by cognitive reserve.
Elkins	2006	US	N= 5,888 (healthy participant)<br><br>N= 3622 with MRI for cross-sectional study	– Ischemic stroke<br>- AGE: >= 65<br>– random sample of individuals identified using Health Care Financing Administration Medicare	Education level	Extensive baseline evaluation:<br>– medical questionnaires,<br>– physical examination<br>– laboratory testing. – Years of formal education (baseline) –	Cross-sectional analysis: Education level strongly associated with performance on the cognitive

			N=1433 for longitudinal study<br><br>Ischemic stroke			Cognitive functions were evaluated annually with the Modified MMSE – 3MS (that includes additional tests of delayed recall and verbal fluency, and the DSST);<br>- cranial MRI scanning	tests. Longitudinal analysis: The results of DSST, measure of psychomotor speed does not support RC theory.
Gil Pagés	2019	EU	N=34<br><br>Ischemic or hemorrhagic stroke	-age over 18 years<br>-diagnosis of first-ever stroke<br>- >12 months after stroke<br>-neuropsychological assessment upon hospital admission<br>-no diagnosis of aphasia<br>-not to be suffering any psychiatric condition<br>- no history of substance abuse.	Static proxies (Education, Occupation)<br>-Dynamic proxies (CRS , CRS-Pre-stroke, CRS-Post-stroke)	NPS assessment:<br>- TMT-, Digit Span forward subtest from WAIS-III, RAVLT, PMR test , Digit Span backward and Letter Number Sequencing from WAIS-III at admission, discharge, and chronic stage<br>-Self-rating scales applied in chronic stage of stroke: CIQ and PCRS (self-rating questionnaires to measure frequency of subjective cognitive complaints.	Study 1: significant mean differences when comparing scores of CRS pre-stroke and CRS post-stroke in the ADLs domain and in the training / formation domain.<br>Study 2: No significant cognitive changes between admission, discharge stage, and discharge-chronic stage of Stroke.<br>Study 3. Significant correlations with CRS-Post stroke in the four domains: ADLs, Training/

							formation, Hobbies and Social Life.
Glymour	2008	US	N=291 Control group,<br><br>N= 272 Ischemic or hemorrhagic stroke	- age >= 45 y;<br>- meeting National Institute of Neurologic Diseases and Stroke criteria for ischemic or nontraumatic hemorrhagic stroke (3<NIHSS<8);<br>- adequate comprehension and expressive capabilities.<br>- base social ties.<br>- live home prior to stroke or return home post stroke.<br>- intact prestroke cognitive functioning.<br>- residence nearby Boston	Socially Supportive Behaviors and Social Ties.	Assessment at baseline (17-20 days after stroke) and 6 months after stroke:<br>–Barrera's Inventory of Socially Supportive Behaviors (only at baseline)<br>– social ties Index (3-point measures: intimate, personal and organizational ties)<br>– Cognitive function: MMSE and 7 NPS test (Digit Span forward, Boston x aphasia, immediate recall of 10 words, the 1 min Animal Naming and TMT A and B).	Good Social ties are an advantage at base line but don't predict better recovery at 6 months. Higher level of emotional support at baseline may promote cognitive resilience while social ties provide cognitive reserve that protects against impaired cognition after stroke (6 moths).
Gonzalez-Fernandez	2011	EU	N=173 Control group,<br><br>N=62 Stroke	-presentation within 24 hours of stroke onset<br>-no history of dementia or previous symptomatic stroke or neurologic disease<br>-no known hearing loss or uncorrected visual impairment<br>-premorbid English proficiency<br>-no contraindications to MRI	-Education dichotomized as less than 12 years of education or 12 or more years of education<br>--SES obtained from census tract data as 2 variables: mean neighborhood household income and family income	-Percent error on 9 language tasks (auditory and written comprehension, naming [oral, written, and tactile], oral reading, oral spelling, written spelling, and repetition<br><br>-Demographic characteristics (age, sex, race) and stroke volume	The percentage of errors for participants with 12 or more years of education was significantly lower for auditory and written comprehension, written naming, oral reading, oral spelling, and written

				-no pregnancy<br>-normal level of consciousness<br>-no hemorrhage on initial scans<br>-no global aphasia<br>-ability to participate in testing		were recorded for adjustment	spelling of fifth grade vocabulary words, even after adjusting for age, sex, stroke volume, and SES.
Ihle	2020	Switzerland	N=897<br><br>Stroke	Individuals who participated in the two waves (W) of the VLV survey, in 2011 and 2017	-CRIq	-TMT in two waves 6 years apart (2011 and 2017)	Older age in W1 significantly predicted a larger increase in TMT completion time from W1 to W2<br>Greater past leisure activity engagement across adulthood significantly predicted a smaller increase in TMT completion time from W1 to W2<br>Education, cognitive demand of jobs, sex, and history of stroke per se did not predict changes in TMT completion time<br>For individuals

						with low past leisure activity engagement, history of stroke in W1 significantly predicted a larger subsequent increase in TMT completion time from W1 to W2.	
Makin	2018	UK	N= 208 (N=157 returned for formal cognitive testing at one to three months post-stroke N=151 returned at one year)<br><br>Ischemic stroke	- clinically-evident lacunar or minor non-lacunar ischemic stroke (NIHSS less than 7)<br>- over 18 y<br>- recorded clinical features and vascular risk factors<br>- MRI<br>- detected stroke sub-type and small vessel disease burden<br>- no pre-stroke dementia or MCI	-Educational attainment (years of education)<br><br>-NART	-ACE-R<br>-mRS<br>-BDI	Lower NART score and older age predicted one-year cognitive impairment more than stroke severity (per point on NIHSS) or vascular risk factors e.g., hypertension.	
Mirza	2016	EU	N=12561<br><br>Stroke or TIA	- no history of dementia, TIA or stroke<br>- available data about education<br>- consent for collection of data at follow-up.	Educational level	- Executive function: LDST, VFT.<br>-Memory: 15-WLT<br>Motor skills<br>Purdue pegboard test-(both hands)<br>-MMSE.	The risk of developing dementia after a stroke or TIA was highest in the low education category, followed by the intermediate education category. The decrease in dementia risk with	

							increasing education was significant only in men.
Ojala-Oksala	2012	US	N=486<br><br>Control group, N=612<br><br>Mild/moderate ischemic stroke	-ischemic stroke;<br>- age between 55 and 85 y;<br>- living in Helsinki;<br>- speaking Finnish language;<br>-Responders after 3 months follow up	Education history	- neuropsychological assessment (MMSE) (n= 409)<br>- MRI Analysis (n=395)<br>- NIHSS scale<br>- MRI 3 moths after (White Matter lesions)	Higher education levels were associated with reduced executive dysfunction, memory impairment, aphasia, visuospatial and constructive deficits, lower MMSE scores, and dementia. Longer educational history was not linked to recurrent strokes but was associated with better post-stroke survival.
Padua	2020	EU	N=75<br><br>Ischemic or hemorrhagic stroke	- stroke;<br>- time since stroke between 2 weeks and 6 months;<br>- age between 40 and 85 years<br>- cognitive and language abilities sufficient to understand the experiments and follow instructions;<br>- no previous stroke history<br>- no behavioral and cognitive disorders	CRIq	- Assessment at baseline (T0) and post-intervention (6 weeks of rehab treatment - 30 sessions (T1).<br>- ARAT<br>- MI<br>- BI CRIq.	Among patient underwent robotic rehabilitation, a moderate correlation between the CRI related to working activities and MI evolution

				- intact visual field<br>- no ankylosis of the upper/lower limbs Fugl-Meyer score >58 (0-66 version)<br>- ability to sign informed consent			
Rosenich	2022	Australia	- N=10<br>- Age = 77.8 ± 10.4 years (range 61-99), 8 male 2 subjects lost to follow-up due to death and severe disability<br><br>Ischemic middle cerebral artery stroke	- Admission with first-ever ischemic stroke<br>- MCA stroke<br>- age >18 years<br>- no other neurological condition(s)	-CRIq).	- Stroke Scale (NIHSS<br>- Fugl-Meyer Assessment at baseline<br>- EEG 3-months post-stroke.	Adding cognitive reserve to the regression model improved its effectiveness despite not being statistically significant. However, cognitive reserve did not improve models for motor impairment at baseline.
Schirmer	2019	US	N=481 Mean age 66.6±14.7 years, 36% were male<br><br>Ischemic stroke	- acute cerebral infarct lesions confirmed by DWI scans obtained within 48 hours of symptom onset<br>- T2-FLAIR sequences available for WMHv analysis.	Effective reserve	- All clinical variables including demographics, past medical history, and vital signs<br>-mRS	An inverse association exists between effective reserve and 90-day mRS scores. Compared to a model without effective reserve, the correlation between predicted and observed mRS
Shin	2020	Asia	N=10636 Interim	- first acute ischemic stroke	- Education<br>- Premorbid	- K-MMSE - Cognitive	Cross-Sectional

			analysis of the Korean Stroke Cohort for Functioning and Rehabilitation<br><br>N=7459 included for cross-sectional analysis<br><br>N=3109 included for longitudinal analysis (N=67 excluded for no follow up)<br><br>Ischemic or hemorrhagic stroke	or intracerebral hemorrhage (confirmed at imaging)<br>- age ≥19 years at stroke onset<br>- onset of symptoms within 7 days before inclusion<br>- no TIA, history of stroke, traumatic intracerebral hemorrhage<br>- Korean nationality	occupation<br>- A composite CR score described in a previous study)	impairment defined as total K-MMSE score <16th percentile (age and education –adjusted).	Analysis: The composite CR score predict cognitive impairment: the higher the composite CR score, the lower the risk. Longitudinal Analysis: A higher composite CR score increased the slope during the first 3 months, but occupation and composite CR score had no significant influence on the second slope.
Skoog	2017	UE	N = 1065<br><br>Cohort 1901–02, N=494<br><br>Cohort 1923–24, N=571<br><br>Stroke	85-year-olds born 1901–02 and 1923–24 obtained from the Swedish Population Register.	Education	- prevalence of dementia (diagnosed according to DSM III-R) in relation to different variables (stroke, birth cohort, sex, marital status and education)<br>- MMSE	Education, stroke, interaction stroke and birth cohort (but not birth cohort) were related to prevalence of dementia. The decline in dementia prevalence was mainly explained by higher education and lower odds for dementia with stroke in later

							born birth cohorts.
Umarova	2021	UE	- N=153 with mean age ± SD, 64.4 ± 13.8 years.<br>- 52 females on average 17.0 ± 18.5 months post-stroke, range 5.0–72.5 months<br><br>Ischemic stroke	- first-ever ischemic stroke in the middle cerebral artery territory. Exclusion criteria in the acute phase were (i) an age over 90 years; (ii) conditions compromising study participation and examination (e.g. low consciousness or arousal level, general MRI contraindications); (iii) pre-stroke neurological or psychiatric conditions compromising data interpretation (pre-stroke cognitive impairment, previous stroke, structural brain lesions besides stroke etc.); and (iv) illiteracy	Education	-MoCA<br>-NIHSS<br>-MRI	Larger brain lesions did not lead to cognitive impairment in younger patients with higher education but did so in younger patients with lower education. Conversely, even small lesions led to poor cognitive outcomes in older patients with lower education, but not in older patients with higher education. Similar interactions were observed for clinical scores of stroke-induced impairment and disability in both the acute and chronic stroke phases.
Umarova	2019	EU	N=10636<br><br>Ischemic stroke	- first-ever territorial ischemic right middle cerebral artery stroke;<br>- no pre-stroke neurological or	- IQ score of Multiple Choise Vocabulary Test<br>- Years of	- NIHSS and Rankin Scale (mRS) assessed at admission (<24h post-stroke) and at discharge (9.5±4.8	Patients with longer educational history had a lower NIHSS score in acute

				psychiatric pathologies<br>- right handedness;<br>- no coma.	education	days post-stroke)<br>NPS: MoCA, Tonic alertness (reaction time in ms to single targets), Working Memory (Digit span forward and backward), Executive functions (word fluency with phonemic and semantic cue), tests for neglect Crystallized intelligence, IQ score of Multiple-Choice Vocabulary Test<br>- MRI examination	stroke. Low level of education was also associated with a higher mRS around 5 years after stroke, independent from age. A beneficial impact of cognitive reserve on disability in acute stroke, is independent from lesion load and age.
Withall	2009	Australia	N=125 Control Group, N=106 Ischemic stroke	- ischemic stroke;<br>- age <=85 years DoC <7 days;<br>- No dementia/alcohol or drug or severe aphasia (Boston test)<br>- Able to inform consensus<br>- 3-6 months from stroke at 1° evaluation<br>- speaking English language	IQ	- Medical assessments<br>- Control of Psychiatric measure DSM-IV, Depression and apathy scales (n=168)<br>- BADL /IADL /MMSE scales (n=125)<br>- control of cognition: NPS battery, premorbid intelligence (NART-R, (n=168)<br>- CT or MRI scan: num and dimension of lesions.	Brain reserve appears to play a protective role, and apathy may be an important moderating variable.
Elayoubi	2022	US	898 subjects; Age: 38–97 years (*M* = 72 years-,	Data from the HRS (RAND, 2016); partecipants who:<br>- reported incident	Social connection and social engagement	Cognitive functions (Word recall task)	Social connection and engagement

			SD = 10); 54% females Severe troke	stroke; - had a cognitive assessment (two data points)			are linked to a slower decline in word recall over time.
Rojas Albert	2022	EU	38 subjects; Age: 47–86; 50% females Ischemic stroke	- First-ever unilateral ischemic stroke; - upper extremity motor deficit involving hand function; - no history of previous neurological or psychiatric illness; - age ≥ 18 years.	Cortical thickness of controlesional cortices (MRI)	Functional outcome (mRS)	Cortical thickness of the contralesional cortices positively linked with outcomes at 3–6 months after the lesion, regardless of initial deficits, lesion volume, or age.
Sadegh ihassan abadi	2022	EU	38 subjects; Age: 47–86; 50% females Acute Ischemic stroke	- First-ever unilateral ischemic stroke; - upper extremity motor deficit involving hand function; - no history of previous neurological or psychiatric illness; - age ≥ 18 years.	Structural MRI cerebellar volumetry	Functional outcome (mRS)	Volumes of cerebellar lobules IV, VI, and VIII positively associated with better outcomes, regardless the severity of initial impairment, age, and lesion volume
Bettger	2014	US	965 ischemic stroke patients who survived to 3 months in the Adherence eValuation After Ischemic stroke – Longitudinal (AVAIL) study Ischemic troke	-Age>17 years hospitalized at a GWTG-Stroke participating hospital with a stroke; - being able to provide informed consent or having a legally authorized representative sign; - having patient data documented in the GWTG-Stroke	SES (education, working status, household income)	Functional outcome (mRS)	SES significantly associated with disability after acute ischemic stroke

	registry.

Legend: ACE-R: Addenbrooke's Cognitive Examination-Revised; ADL/BADL: Activity Daily Living; APT: Attention Process training; ARAT: Action Research Arm Test; BAA: Bilingual Aphasia Adults; BAT: Bilingual Aphasia Test; BDI: Beck Depression Inventory; BHA: Bilingual Healthy Adults; BI: Barthel index; BNT: Boston Naming test; CDR: Clinical Dementia Rating scale; CIQ: Community Integration Questionnaire; CRS Cognitive Reserve Scale; CNS: Central Nervous System; CRIq: Cognitive Reserve Index; DoC: Disorder of consciousness; *DRS: Disability Rating Scale; DSM: Diagnostic and Statistical Manual of Mental Disorders; DSST: Digit Symbol Substitution Test; DWI: diffusion weighted imaging; EEG: Electroencephalography; GCS: Glasgow Coma Scale; GOS-E: Glasgow Outcome Scale- Extended; HADS: Hospital Anxiety and Depression Scale; K-MMSE: Korean mini-mental state examination; LCF: Level of Cognitive Functioning; LDST: letter-digit substitution task; LUQ: Language Use Questionnaire; mBI: modified Barthel index; MAA: Monolingual Aphasia Adults; MCA: middle cerebral artery; M- DASS-21:Malaysian Depression and Anxiety Screening Scales 21; MCI: Mild Cognitive Impairment; MHA: Monolingual Healthy Adults; MoCA: Montreal Cognitive Assessment; Modified MMSE: 3MS Modified Mini mental State Examination; MRI: Magnetic Resonance Imaging; mRS: modified Rankin Scale; NART: National Adult Reading Test; NIHSS: National Institute of Health Stroke Scale; NLTT: Non-linguistic Triad task; PCRS: Patient Competency Rating Scale; PMR test: Spanish version of FAS test- phonemic verbal fluency; RBMT: Rivermead Behavioural Memory Test; SES: Socio-economic status; Tele-GEMS: Tele-Global Examination of Mental State; TBI: Traumatic Brain Injury; TIA: Transient Ischemic Attack; TMT: trail Making Test; VLV: Vivre-Leben-Vivere; WAIS: Wechsler Adult Intelligence Scale; T2-FLAIR: T2 fluid- attenuated inversion recovery; VFT: verbal fluency test; WHOQOL BREF-2: World Health Organization-Quality of Life- Bref-2.; WMH: White Matter Hyperintensities; 15-WLT:15-word verbal learning test.*
